# RETRACTION: A Novel Skin Lesion Prediction and Classification Technique: ViT‐GradCAM

**DOI:** 10.1111/srt.70154

**Published:** 2025-01-07

M. Shafiq, K. Aggarwal, J. Jayachandran, G. Srinivasan, R. Boddu, and A. Alemayehu, “A Novel Skin Lesion Prediction and Classification Technique: ViT‐GradCAM,” *Skin Research and Technology* 30, no. 9 (2024): e70040, https://doi.org/10.1111/srt.70040.

The above article, published online on 2 September 2024 in Wiley Online Library (wileyonlinelibrary.com), has been retracted by agreement between *Skin Research and Technology*; and John Wiley & Sons Ltd. The article was submitted as part of a guest‐edited special issue. Following publication, it has come to the attention of the journal that the article was accepted solely on the basis of compromised editorial handling and peer review processes. Furthermore, a substantial portion of the article's results and conclusions is unsupported, as part of the described findings could not have been produced with the presented methodology.

Therefore, the decision to retract this article was taken.

